# Dissecting the Hormonal Signaling Landscape in Castration-Resistant Prostate Cancer

**DOI:** 10.3390/cells10051133

**Published:** 2021-05-07

**Authors:** Fabrizio Fontana, Patrizia Limonta

**Affiliations:** Department of Pharmacological and Biomolecular Sciences, Università degli Studi di Milano, 20133 Milano, Italy; fabrizio.fontana@unimi.it

**Keywords:** castration-resistant prostate cancer, androgens, androgen receptors, AR, gonadotropin-releasing hormone, GnRH, gonadotropin-releasing hormone receptors, GnRH-R

## Abstract

Understanding the molecular mechanisms underlying prostate cancer (PCa) progression towards its most aggressive, castration-resistant (CRPC) stage is urgently needed to improve the therapeutic options for this almost incurable pathology. Interestingly, CRPC is known to be characterized by a peculiar hormonal landscape. It is now well established that the androgen/androgen receptor (AR) axis is still active in CRPC cells. The persistent activity of this axis in PCa progression has been shown to be related to different mechanisms, such as intratumoral androgen synthesis, AR amplification and mutations, AR mRNA alternative splicing, increased expression/activity of AR-related transcription factors and coregulators. The hypothalamic gonadotropin-releasing hormone (GnRH), by binding to its specific receptors (GnRH-Rs) at the pituitary level, plays a pivotal role in the regulation of the reproductive functions. GnRH and GnRH-R are also expressed in different types of tumors, including PCa. Specifically, it has been demonstrated that, in CRPC cells, the activation of GnRH-Rs is associated with a significant antiproliferative/proapoptotic, antimetastatic and antiangiogenic activity. This antitumor activity is mainly mediated by the GnRH-R-associated Gαi/cAMP signaling pathway. In this review, we dissect the molecular mechanisms underlying the role of the androgen/AR and GnRH/GnRH-R axes in CRPC progression and the possible therapeutic implications.

## 1. Introduction

Prostate cancer (PCa) still remains the second leading cause of cancer-related deaths in Western countries, although a higher survival rate and a long-term decline in mortality have been recently reported [1]. Most PCas are androgen-dependent in their early stage, and androgen deprivation therapy (ADT), aimed to reduce the circulating levels of testosterone and achieved by chemical castration, still represents the standard care of treatment [2,3,4]. Gonadotropin-releasing hormone (GnRH) analogs (agonists and antagonists), responsible for the suppression of testicular androgen production, are often associated with inhibitors of androgen receptor (AR) activity to obtain a maximal androgen deprivation condition (combined androgen blockade, CAB) [5,6]. Unfortunately, within 2–3 years, most patients progress towards the so called castration-resistant prostate cancer (CRPC) stage, characterized by tumor growth, even in the presence of castration levels of circulating androgens [7,8].

It is now well established that the androgen/androgen receptor (AR) axis remains a key player in the growth of CRPC [9,10,11,12,13]. Moreover, not only steroids but also peptide hormones and their receptors are deeply involved in the process of PCa progression. Gonadotropin-releasing hormone (GnRH) is the hypothalamic decapeptide known to be a key player in the functions of the pituitary gonadal axis through the activation of its pituitary receptor (GnRH-R) [14,15,16,17,18,19]. GnRH and GnRH-Rs are also expressed in different cancer cells and tissues, including PCa, both androgen-dependent and castration-resistant; the activation of these receptors is associated with a significant antitumor activity [20,21,22,23,24,25,26,27,28,29,30,31,32,33,34].

## 2. The Androgen/AR Axis in CRPC

Second-line antiandrogen therapy is indicated for the treatment of CRPC patients, supporting the idea that the androgen/AR axis is still active in this progression phase of the tumor [35,36,37,38,39,40,41]. To date, several mechanisms involving a persistent activity of the androgen/AR axis in CRPC have been elucidated [12,13,42,43,44].

### 2.1. Intratumoral Synthesis of Androgens

About two decades ago, it was reported that, after ADT therapy, intratumoral levels of androgens remain high in spite of serum castration levels of testosterone [45]. This observation suggested that circulating adrenal androgens might be uptaken by CRPC cells to be converted to testosterone and dihydrotestosterone (DHT). Subsequently, it was demonstrated that CRPC tissues overexpress both 3β-HSD, the enzyme responsible for the conversion of the adrenal steroid dehydroepiandrosterone (DHEA) to androstenedione, and AKR1C3 (17β-HSD), the enzyme involved in the conversion of androstenedione to testosterone and DHT and, therefore, to active androgens [46,47,48]. Transcription factors regulating the expression of genes involved in androgen biosynthesis are also expressed in CRPC cells [49].

It has also been suggested that, in CRPC tissues, androgens can be synthesized from cholesterol; however, this issue is still a matter of debate [48,50]. CYP17A1, the enzyme involved in the synthesis of DHEA and androstenedione, is expressed not only in the adrenal gland but also in PCa tissue [47,50]; treatment options for CRPC patients presently include abiraterone, a specific inhibitor of CYP17A1 activity [51,52,53].

### 2.2. Androgen Receptor Amplification

CRPC tissues (about 80%) from patients who progressed after ADT express high levels of AR; 30–50% of these tissues were reported to carry AR amplification, due to the presence of a high *AR* gene copy number [54,55,56]. AR amplification was also detected in circulating tumor cells (CTCs) from patients with metastatic PCa [57]. The overexpressed receptor is sensitive to low levels of androgens, thus allowing PCa cells to progress towards the CRPC stage. Interestingly, AR amplification has been found to be more common in enzalutamide than in abiraterone-resistant patients [58,59].

### 2.3. Androgen Receptor Mutations

The *AR* gene, belonging to the steroid hormone receptor superfamily, composed of eight exons, is mapped on chromosome Xp11-12 and encodes a 110 kDa (920 amino acids) protein. The full-length AR protein consists of three domains: the NH_2_-terminal transactivation domain (NTD, encoded by exon 1), the central and conserved DNA-binding domain (DBD, encoded by exons 2 and 3), a flexible hinge region containing a nuclear localization signal and the COOH-terminal ligand-binding domain (LBD, encoded by exons 4–8). The NTD domain contains two trinucleotide repeats that encode polyglutamine and polyglycine tracts. The interaction between NTD and LBD is necessary for the receptor transcriptional activity. In the absence of androgens, the AR is located in the cytoplasm, where it is present in an inactive conformation associated with heat shock proteins. Once bound by androgens (testosterone, DHT), the receptor translocates into the nucleus, where it is activated through dimerization, recruitments of coregulatory/epigenetic factors and stimulation of specific target genes [12,13,43,60,61,62].

Gain-of-function mutations of the AR are quite frequent (about 50%) in CRPC patients after antiandrogen therapy; these are usually single point mutations occurring mostly in the LBD of the receptor [63,64]. The T878A mutation, with alanine being substituted by threonine, was shown to confer resistance to both first- and second-generation antiandrogens (enzalutamide, apalutamide, darolutamide); similar observations were reported for the H875Y, W742C and F876L mutations [58,65,66,67,68,69]. Interestingly, by binding to these mutant receptors, antiandrogens can induce their activation, thus behaving as AR agonists; moreover, these mutations were also shown to be activated by different steroids, such as adrenal androgens, progesterone and estrogens [70,71,72]. Consequently, AR mutations are responsible for the continuous activation of the receptor even in the presence of low circulating androgen levels after ADT therapy, thus playing a key role in tumor progression. Some gain of function mutations were shown to favor the recruitment of coregulators to the promoter region of AR target genes, thus increasing the transcriptional activity of this receptor [73,74]. 

Recently, mutant *AR* receptor genes were reported to be easily detectable in cell-free DNA (cfDNA) obtained from CRPC patients. These data strongly support that the presence of specific circulating mutant ARs can represent a useful biomarker in terms of personalized therapy in CRPC [58,75,76,77].

**Figure 1:**
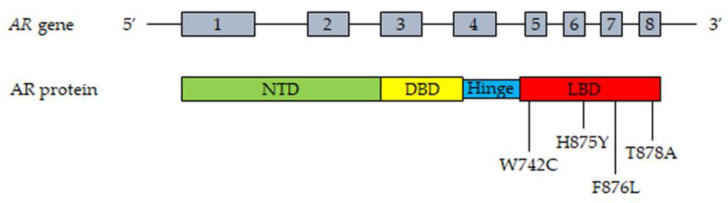
Most frequent mutations of AR detected in tissues and plasma cfDNA from CRPC patients. Upper part, representation of the *AR* gene structure. Lower part, representation of the AR protein structure with the most frequently mutated amino acids. T878A, alanine substituted by threonine; H875Y, tyrosine substituted by histidine; W742C, cysteine substituted by triptophan; F876L, leucine substituted by phenylalanine. Abbreviations: AR, androgen receptor; NTD, NH_2_-terminal transactivation domain; DBD, DNA-binding domain; LBD, ligand-binding domain.

The most frequent mutations of the AR in CRPC patient tissues and plasma (cfDNA) are reported in Figure 1.

### 2.4. Androgen Receptor Splice Variants

During the last two decades, it has become increasingly clear that, in PCa cells, the *AR* gene can undergo alternative splicing, giving rise to different splice variants (AR-Vs) [78,79,80,81,82]. Most of these variants lack the LBD but maintain the ability to enter the nucleus and to bind specific DNA response elements in a ligand-independent manner. Specifically, AR-V7 is truncated at the end of exon 3, but it has been demonstrated to be localized at the nuclear level and to retain constitutive transcriptional activity in the absence of the ligand [83]. The expression of AR-V7 was found to be associated with the development of drug resistance (enzalutamide, abiraterone) and aggressive behavior in CRPC cells [84]. In nude mice harboring human CRPC cell xenografts, treatment with abiraterone significantly increased AR-V7 expression while AR-V7 overexpression promoted tumor growth and invasiveness [85,86]; in line with this observation, targeting this receptor variant was reported to suppress tumor growth and to confer sensitivity to antiandrogens (enzalutamide) [87]. In humans, the expression of AR-V7 was observed in tumor biopsies from CRPC patients and significantly correlated with tumor progression and short survival [88,89,90,91,92]. High levels of AR-V7 could also be detected in extracellular vesicles purified from the plasma of CRPC patients, as well as in circulating tumor cells (CTCs) from enzalutamide- or abiraterone-resistant patients, and were found to be associated with a poor prognosis [93,94,95,96,97].

Additional transcript variants are generated by alternative splicing of the *AR* gene in CRPC [13,98,99]. In particular, the ARv567es splice variant originates from the loss of exons 5–7, encoding the LBD, but it conserves the hinge region of exon 4 involved in the nuclear localization of the receptor isoform, thus supporting its constitutive activity irrespective of the presence of the ligand; its expression was shown to increase in tissue biopsies after ADT therapy, and to correlate with outcomes to taxane therapy in CTCs, in PCa patients [88,100,101].

Mechanistically, the AR-Vs can form homodimers or heterodimers (by combining with other variants), or they can dimerize with the full-length AR. At the nuclear level, the dimers bind to response elements in the promoter region of specific downstream genes (either unique or canonical AR-regulated genes), thus modulating their expression and promoting the development of CRPC [13,42,89,91,102].

Based on these observations, targeting the AR-Vs and their signaling pathways might represent a novel and effective therapeutic strategy for the treatment of CRPC patients.

### 2.5. Androgen Receptor: Transcription Factors and Coregulators

AR-mediated gene transcription requires the interaction of the receptor with different coregulators (such as the Steroid Receptor Coactivators, SRCs) and transcription factors (such as GATA2 and FOXA1) [103,104,105,106]. 

The transcriptional activity of AR requires the recruitment and cooperation of transcription factors. Among these, the GATA family of transcription factors, consisting of six members, was reported to be involved in the AR-mediated signaling in CRPC cells [42,43,106]. In particular, GATA2 pioneer transcription factors were shown to be involved in the androgen-related regulation of PSA expression in CRPC cells; moreover, GATA protein consensus DNA sequences were observed in the AR binding regions of androgen-regulated genes in these cells, supporting their cooperation with the receptor in mediating androgen effects [106,107]. This factor was also demonstrated to be a key regulator of the transcriptional activity of AR-Vs in CRPC cells [43].

FOXA-1 (forkhead box A1) is another pioneer transcription factor involved in AR-promoted gene transcription. It was demonstrated to play a key role in AR-mediated tumor growth and progression in CRPC cells [108]. Mechanistically, FOXA1 binds to AR and the transcription factor HOXB13 at the cytoplasmic level; then, the FOXA1-AR-HOXB13 complex translocates into the nucleus where it binds, with the cooperation of GATA2, to specific DNA sequences. FOXA1, HOXB13 and GATA2 open compacted chromatin, increasing the accessibility of these DNA regions to additional transcription factors, thus promoting AR transcriptional activity and the expression of AR-regulated genes [42,108,109,110].

Coregulators modulate the activity of several proteins in the transcription complex through chemical modifications and are also involved in the recruitment of general transcription factors associated with RNA polymerase II to the constitutive promoter of target genes [106,111]. Specifically, the p160 steroid receptor coactivators (SRC-1, SRC-2 and SRC-3) promote the formation of a complex between AR enhancer sequences and the promoter region of androgen target genes, thus favoring AR transcriptional activity [112]. SRCs expression was found to positively correlate with PCa progression and recurrence [113,114,115]. Importantly, in PCa cells, SRC-2 was reported to interact with AR at the nuclear level to increase the sensitivity of cancer cells to androgens and to enhance the ligand-independent transcription of AR target genes [116,117]. Similarly, the AR coactivator MAGE-11 (melanoma antigen gene protein-A11) was shown to be overexpressed in CRPC cells as a consequence of the hypomethylation of CpG islands in its promoter region, providing an additional mechanism for the increased AR signaling in CRPC [118].

Taken together, these observations support the notion that the interaction of AR with specific transcription factors and coregulators plays a key role in promoting PCa growth and progression. These mechanisms are presently considered a possible molecular target for novel therapeutic approaches for CRPC.

**Figure 2:**
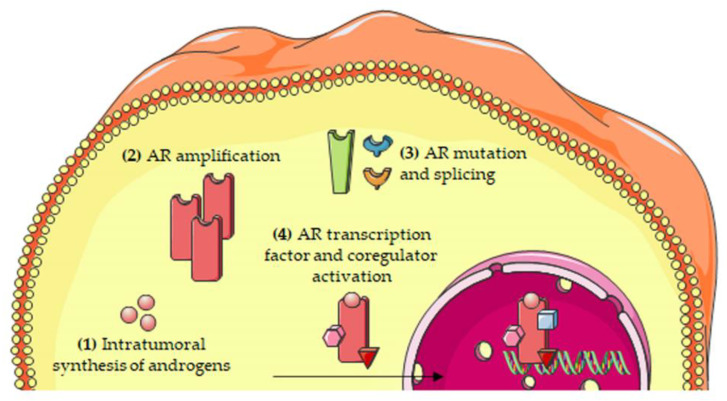
Molecular mechanisms involved in the persistent activity of the androgen/AR axis in CRPC cells. The persistent activation of the androgen/AR axis has been attributed to several mechanisms, including: (**1**) intratumoral synthesis of androgens; (**2**) AR amplification; (**3**) AR mutations and alternative splicing; and (**4**) increased expression/activity of transcription factors and coregulators of the AR. Abbreviations: AR, androgen receptor.

The most relevant molecular mechanisms underlying the persistent activity of the androgen/AR axis in CRPC cells are summarized in Figure 2.

## 3. The GnRH/GnRH-R Axis in CRPC

GnRH agonists (goserelin, triptorelin, histrelin, leuprolide) bind to pituitary GnRH-Rs and, after an initial stimulation (the so called flare event), induce their down-regulation and desensitization leading to the suppression of gonadotropins (LH and FSH), and subsequently, of testosterone secretion [119,120]. To avoid the flare effect, as well as the GnRH-associated side effects (metabolic dysfunction and cardiovascular diseases), GnRH antagonists (cetrorelix, degarelix, abarelix, ozarelix, ganirelix, relugolix) were developed. These compounds act by competitively binding to the pituitary GnRH-Rs, thus immediately suppressing LH and FSH secretion; they were also reported to decrease FSH release for a longer time period and to lower levels than GnRH agonists [121]. Thus, GnRH agonists and antagonists still represent the standard ADT therapy for patients with metastatic PCa [122,123,124].

In the last three decades, it has been widely demonstrated that GnRH and GnRH-Rs are expressed also in tumor tissues, including PCa and, specifically, CRPC [21,23,24,25,26,28,29,32,33,125,126,127]. This intratumor GnRH-GnRH-R axis is associated with a significant anticancer activity, supporting the notion that it may represent an effective target for novel anticancer strategies.

### 3.1. Gonadotropin-Releasing Hormone

Hypothalamic GnRH (pGlu^1^-His^2^-Trp^3^-Ser^4^-Tyr^5^-Gly^6^-Leu^7^-Arg^8^-Pro^9^-Gly^10^-NH_2_) is a decapeptide synthesized in a small number of neurons and released in a pulsatile way into the portal blood vessels, through which it reaches the gonadotrope cells at the pituitary level. Here, it binds to its specific receptors, GnRH-Rs, to stimulate gonadotropin secretion and, consequently, gonadal androgen production [14,15]. The N-terminal (Glp-His-Trp-Ser) and the C-terminal (Pro-Gly-NH_2_) domains of the decapeptide are essential for its binding to GnRH-Rs. 

In addition to the classical form of GnRH (also called GnRH-I), other forms of the peptide have been identified. The isoform II (GnRH-II) has been observed in most vertebrates [128]. It is a decapeptide whose amino acid sequence differs from that of GnRH in the positions 5, 7 and 8 (His^5^, Trp^7^, Tyr^8^), known to be involved in the biological functions of the neurohormone. On the other hand, GnRH-II conserves the amino acid sequence of GnRH in both the N- and C-terminal domains, supporting that it may recognize, bind and activate the same receptors. The presence of a receptor specific for GnRH-II in vertebrates is still a controversial issue [18,19,128,129,130,131,132,133,134]. A third form of GnRH, GnRH-III, was identified in sea lamprey (*Petromyzon marinus*); its structure differs from that of GnRH in amino acids 5–8 (His^5^, Asp^6^, Trp^7^, Lys^8^); this isoform was shown to possess a very low gonadotropin-releasing effect in vertebrates [19,135,136].

GnRH was reported to be expressed in different types of cancer cells. Specifically, GnRH mRNA expression and immunoreactivity were identified in breast, ovarian, endometrial as well as in PCa cells, both androgen-dependent and castration-resistant. In tumor cells, including CRPC cells, this peptide was shown to be endowed with a significant antiproliferative/proapoptotic biological activity [137,138,139,140,141].

### 3.2. Gonadotropin-Releasing Hormone Receptors: Molecular Structure

The molecular structure of the pituitary GnRH-R, belonging to the GPCR (G protein coupled receptor) receptor family, was identified by Kakar et al., in 1992 [142]. Its gene is located on chromosome 4q13.2 and encodes for a 328 amino acid protein consisting of an extracellular N-terminal domain, a seven helical transmembrane-spanning domain and a uniquely short (1–2 amino acids) intracellular C-terminal domain [133,143,144,145]. This last domain is responsible for its desensitization triggered by GnRH agonists [146]. 

Endogenous GnRH, as well as its synthetic agonists, by binding to this receptor, triggers the recruitment/activation of the Gαq/11 subunit of the G protein complex, thus activating the downstream effector phospholipase Cβ (PLCβ). PLCβ, in turn, catalyzes the hydrolysis of phosphatidylinositol 4,5-biphosphate (PIP_2_) into the two second messengers, inositol 1,4,5-triphosphate (IP_3_) and diacylglycerol (DG), leading to protein kinase C (PKC) activation and increased cytoplasmic levels of Ca^2+^, respectively. Both PKC and Ca^2+^ trigger specific downstream signaling pathways involving proteins of the MAPK cascade (ERK1/2, JNK, p38MAPK), finally mediating the biological activity of the receptor ligands, i.e., expression and secretion of the two gonadotropins [27,147,148,149,150,151]. 

As discussed above, it is now well established that GnRH-Rs are expressed also in different tumor cells and tissues, including PCa, and specifically CRPC [20,21,22,23,24,25,26,28,29,30,31,32,33,34,152]. 

The binding affinity of GnRH analogs for these receptors was first investigated, leading to contrasting results. Two classes of GnRH-binding sites, one with low affinity and one with high affinity, were found in human PCa cells, as well as in the Dunning R3327 prostatic adenocarcinoma in rats [153,154]. On the other hand, in our laboratory, we could observe the presence of one single class of low affinity GnRH-binding sites in PCa cells, both androgen-dependent and castration-resistant [139,155], while the presence of a single class of high affinity GnRH-binding sites was reported in PC3 CRPC cells and in Dunning R3327 prostatic tumor tissue [156,157].

At the molecular level, we demonstrated that a GnRH-R, with the same mRNA and protein size with the gonadotrope receptors, is expressed in human androgen-dependent and CRPC cells [21,137,158,159]; these observations were further confirmed by studies performed in rat Dunning R3327 prostatic adenocarcinoma and in human PCa cells and tissue biopsies [160,161,162,163].

The presence of a specific receptor for GnRH-II (GnRH-II-R) in human tissues has been widely investigated; however, this receptor still must be cloned and sequenced. It has been proposed that the GnRH-II-R might correspond to a five transmembrane domain protein, which lacks the transmembrane regions 1 and 2 [132]. Another variant form of this receptor was also reported to be expressed in human tissues and suggested to be nonfunctional due to the presence of a frameshift in exon 1 and a stop codon in exon 2 [164]; on the other hand, this receptor variant was reported to be present in sperm and testis and to be endowed with a functional role in spermatogenesis [165].

The presence of the GnRH-II-R was also investigated in human cancers. Emons and coworkers reported the presence of a functional GnRH-II-R in tumors of the female reproductive system (ovary, endometrium) [29,32,33,166]. On the other hand, we demonstrated that, in CRPC cells, the antiproliferative activity of GnRH-II is mediated by the classical form of the GnRH-R [167]; similar observations were reported by Kim and collaborators [168]. Taken together, these observations support the claim that the presence of a functional GnRH-II-R in human tumor tissues is still a controversial issue.

### 3.3. Gonadotropin-Releasing Hormone Receptors: Antiproliferative Activity

The activation of locally expressed GnRH-Rs, by means of GnRH agonists, was widely reported to induce antitumor/proapoptotic effects in PCa cells. Specifically, we demonstrated that GnRH agonists, significantly and dose-dependently, reduce the proliferation of both androgen-dependent (LNCaP) and castration-resistant (PC3, DU145) human PCa cells, both in vitro and in vivo [21,22,23,139,155,169,170]. GnRH agonists were also shown to suppress the growth of the rat Dunning R-3327 model of PCa as well as of primary human PCa cell cultures [156,171]. This activity was found to be mediated by the induction of G2/M phase cell cycle arrest [172,173]. These experimental data are supported by the observation that, in CRPC patients, GnRH agonist-based therapy is associated with a longer disease-specific survival in the presence of a high expression of GnRHRs [174].

GnRH-R activation was further reported to be associated with proapoptotic effects [175,176]. In particular, in CRPC cells, GnRH agonists were shown to trigger apoptosis-related molecular events through the down-regulation of the PI3K/AKT signaling pathway, leading to the activation of the downstream JNK kinase, and MEK/ERK kinase activity [177,178]. In our laboratory, we found that GnRH agonists can sensitize, and resensitize, CRPC cells to the proapoptotic activity of the chemotherapeutic agent docetaxel [179]. In line with these observations, cleaved caspase-8 and -3, but not -9, and increased expression and phosphorylation of p53, were reported to increase in primary cell cultures from human PCa samples, supporting the idea that the extrinsic (but not intrinsic) apoptosis pathway is involved in the antitumor activity of GnRH agonists [171,173,180,181]. The different cell context-dependent biological effects of GnRH agonists at the pituitary vs. PCa cell level was suggested to be related to a transient vs. sustained activation of the intracellular signaling pathway (PKC/MAPK) in gonadotropes vs. cancer cells [176,182].

It is now well known that GnRH-R activation interferes with the protumor activity of growth factors and their locally expressed receptors in cancer, and, specifically, in PCa cells [21,22,23,24,29,158,183,184].

The insulin-like growth factor (IGF) signaling axis, composed of two receptors (IGF-IR and IGF-IIR) and their specific ligands (IGF-I and IGF-II), was widely shown to be up-regulated in PCa cells and tissues [185,186,187] and is now considered an effective molecular target for PCa therapy [188,189,190]. We reported that, in CRPC cells, GnRH agonists interfere with the protumoral activity of IGF-I, by reducing IGF-IR expression and activation (i.e., tyrosine phosphorylation) [21,159]. Ahearn and coworkers observed that IGF-IR expression positively correlates with tumor stage in human PCa biopsies [191]; a positive correlation was also observed between the expression of IGF-II and the histologic differentiation and pathologic stage, lymph node metastasis and serum PSA (prostate-specific antigen) levels in hormone-treated PCa patients [192].

The EGF/EGF-R (epidermal growth factor and its receptor) signaling pathway is also deeply involved in PCa growth and progression [184,193,194,195]. We demonstrated that GnRH agonists abrogate the stimulatory effects of EGF on CRPC cell proliferation, in vitro and in vivo, by reducing the expression of EGF-Rs and its downstream transcription factor c-fos [196]. In line with these data, GnRH agonists were found to interfere with the mitogenic activity of EGF and its intracellular signaling pathways in androgen-dependent as well as in CRPC cells [197,198,199].

Based on the undesired initial flare effect triggered by GnRH agonists, GnRH antagonists, able to compete with the binding of the endogenous decapeptide to its pituitary receptors, were subsequently developed, and it was expected that these compounds might act as GnRH-R antagonists also in tumors. Surprisingly, it was widely demonstrated that GnRH antagonists act as agonists in cancer cells, including PCa cells, exerting a significant antiproliferative/proapoptotic activity both in vitro and in vivo [24,25,26,29,200,201,202]. In line with these observations, Castellon and coworkers reported that the GnRH antagonist cetrorelix induces an antiproliferative and proapoptotic effect in primary cell cultures from human prostate carcinoma [171]. Sakai et al. analyzed the effects of the GnRH antagonist degarelix on the growth of androgen-dependent and CRPC cells, as well as on VCaP cells derived from a patient with hormone-refractory PCa. They found that degarelix reduces cell viability by triggering apoptosis, both extrinsic and intrinsic, as indicated by increased caspase 3/7, 8 and 9 levels [203]. Interestingly, Cucchiara and coworkers recently reported that degarelix significantly decreases the proliferation of C4–2B MDVR (enzalutamide resistant, expressing high levels of the AR splice variant AR-V7, after a long exposure to the AR antagonist) PCa cells. This antitumor activity was found to be related to a decreased expression of the AR variant, both at the mRNA and at the protein level [204]. Since the GnRH antagonist had a greater impact on protein than on mRNA levels, the authors suggested that this compound might act by triggering a protein degradation through the ubiquitin proteasome system [204,205,206]. Different molecules such as ASF/SF, JMJD1A, U2AF65, hnRNPA1 and HoxB13 were demonstrated to be involved in AR splicing mechanisms; however, whether degarelix might affect their activity still remains to be investigated [207,208,209,210].

To explain the agonistic behavior of GnRH antagonists at the level of tumor cells, Millar and coworkers proposed that these receptors may adopt different conformations according to the cell context in which they are expressed, thus selectively binding to the different GnRH analogs (the “ligand-induce selective signaling” theory) [211]. 

In addition to the classical form of GnRH, its GnRH-II isoform has also been reported to be expressed in tumors, including PCa [21,29,31,212]. Similar to GnRH, GnRH-II was found to be endowed with a significant antiproliferative/proapoptotic activity in both androgen-dependent and CRPC cells [21,212,213]. Interestingly, the classical form of the GnRH-R was demonstrated to mediate the antitumor effects of GnRH-II in these cells [167]. 

### 3.4. Gonadotropin-Releasing Hormone Receptors: Antimetastatic and Antiangiogenic Activity

CRPC is frequently associated with the development of metastasis, specifically at the bone, lymph node and visceral level; a better understanding of the molecular mechanisms involved in the metastatic events associated with PCa progression might help identify novel biomarkers and possible targets to increase the therapeutic approaches against this almost untreatable disease [12,214,215,216,217].

A remodeling of the intracellular cytoskeleton as well as of the extracellular matrix is deeply involved in the motility and invasive behavior of cancer cells associated with the tumor metastatic spread. We demonstrated that, in CRPC cells, GnRH-R activation significantly reduces both cell migratory and invasive behavior. Moreover, GnRH agonists interfere with the prometastatic activity of IGF-I by affecting cell morphology, cytoskeleton organization and the expression of the αvβ3 integrin, involved in the cell to extracellular matrix adhesion in PCa tissues [218]. 

In line with these data, it was demonstrated that not only GnRH but also the GnRH-II isoform inhibits CRPC cell motility through the remodeling of actin cytoskeleton [219].

Dondi and coworkers reported that, in DU145 and PC3 CRPC cells, GnRH analogs inhibit the activity of the plasminogen activator (PA) system, implicated in the local degradation of the extracellular matrix. Specifically, they showed that both the GnRH agonist leuprolide and the antagonist cetrorelix decrease cell motility and invasiveness by reducing the enzymatic activity and the secretion of uPA (urokinase-type PA), while increasing the expression of the PA inhibitor PAI-1 [220]. In line with these data, cetrorelix was reported to suppress DU145 cell invasiveness by decreasing the expression of proteins involved in cell-to-cell adhesion molecules (i.e., E-cadherin, α- and β-catenin) [221].

It is well known that metastasis is a complex process that involves the cooperative actions of different cancer cell subpopulations, in which cancer stem cells would be responsible for the final step of colonizing premetastatic niches. Cancer stem cells are also deeply involved in the mechanisms of drug resistance, being able to avoid the effects of standard antitumor therapies [222]. Recently, Contreras and coworkers isolated and characterized a cell subpopulation with stem-like properties from explants of human prostate tumors and found that these cells do not express the GnRH-R [34,223].

Angiogenesis is the process by which new vascular vessels form from the pre-existing vasculature. In tumors, the formation of new blood vessels is necessary to provide an appropriate blood supply to support cell viability and proliferation. Hence, this process plays a key role in tumor progression and is now recognized as one of the hallmarks of cancer [224,225]. Vascular endothelial growth factor (VEGF) is an essential factor for vascular endothelial cells; its expression is up-regulated in most cancers, and its crucial role in tumor angiogenesis is well defined [226,227,228,229,230,231]. Among the VEGF isoforms, VEGF-A was shown to play a key role in PCa angiogenesis [232,233,234]. VEGF-A is overexpressed in PCa, and high levels of this growth factor are associated with the presence of metastasis and a poorer prognosis [235,236]. Furthermore, in PCa, a high expression of VEGF-A was observed not only in endothelial cells, but also in tumor cells [235].

In our laboratory, we observed that GnRH-Rs are expressed in HUVEC (human umbilical vein endothelial) cells; GnRH agonists reduce HUVEC cell proliferation and counteract the effects of VEGF-A on their ability to form capillary-like tubes [237]. Recently, a bifunctional fusion protein (LMRAP), consisting of a GnRH Fc fragment and an integrin targeting peptide, was developed as a new strategy for the therapy of GnRH-R expressing tumors. The antitumor activity of this protein was assessed in different cancer cells lines, including CRPC cells, in vitro and in vivo. It was reported that this protein significantly inhibits tumor growth and angiogenesis [238].

### 3.5. Gonadotropin-Releasing Hormone Receptors: Signal Transduction

The inhibitory effects of GnRH-R activation on tumor growth and progression suggested that, in cancer cells, these receptors might be coupled with intracellular signaling mechanisms different from those found at the pituitary level. It is now well accepted that, while GnRH-Rs expressed on the gonadotrope cell membranes are associated with the Gαq/PLC/PKC signaling pathway, in cancer cells, these receptors are mainly coupled with the Gαi/cAMP/PKA cascade of transduction mechanisms [21,29,31,148,211,239,240]. 

We demonstrated that, in PCa and, specifically, CRPC cells, GnRH-R activation significantly interferes with the forskolin-induced increase in cytoplasmic cAMP levels while pertussis toxin counteracts the antiproliferative effects of GnRH analogs [152], suggesting that the Gαi signaling pathway is involved in the anticancer activity of these compounds. In these cells, by decreasing cAMP levels, GnRH-R ligands trigger the activation of a PTP (phosphotyrosine phosphatase), an enzyme responsible for the dephosphorylation (i.e., inactivation) of growth factor receptors. It is well established that GnRH ligands prevent the activity of growth factors, such as EGF and IGF-I, thus leading to the suppression of the expression/activity of their intracellular signaling mediators (i.e., ERK1/2, PI3K/AKT and *c-fos*) and, consequently, of their protumoral effects [196,218,239]. In human benign prostatic hyperplasia (BPH-1) cells, GnRH-R ligands (agonists and antagonists) stimulate the Gαi-mediated activation of the p38MAPK and JNK kinases [240]. The JNK/Jun signaling pathway, triggered by AKT inhibition and subsequent stabilization/activation of its upstream regulator MLK3 (mixed-lineage kinase 3), was also shown to be involved in the anticancer activity of GnRH analogs in PCa cells [28,177]. Similar observations were reported in cancer cells of the female reproductive system [29,241,242]. 

Naor’s laboratory widely demonstrated that the Gαq/PLC/PKC pathway is also involved in the antiproliferative/proapoptotic activity of GnRH analogs in cancer cells [243]. It was found that different PKC isoforms (PKCα, PKCβII and PKCε) are present in gonadotropes as well as in PCa cells, despite being at a different level of expression. Moreover, in CRPC cells, GnRH agonists induce a sustained activation of the PKC/MAPK (p38MAPK and JNK) signaling cascade [182]. In particular, a c-Src-mediated signal and a reduction of AKT activity were found to be involved in the activation of the MLK3/JNK axis [176]. 

Taken together, these data strongly support the notion that different GnRH-R-associated signaling pathways are involved in the opposite effects of GnRH analogs in pituitary vs. CRPC cells.

**Figure 3:**
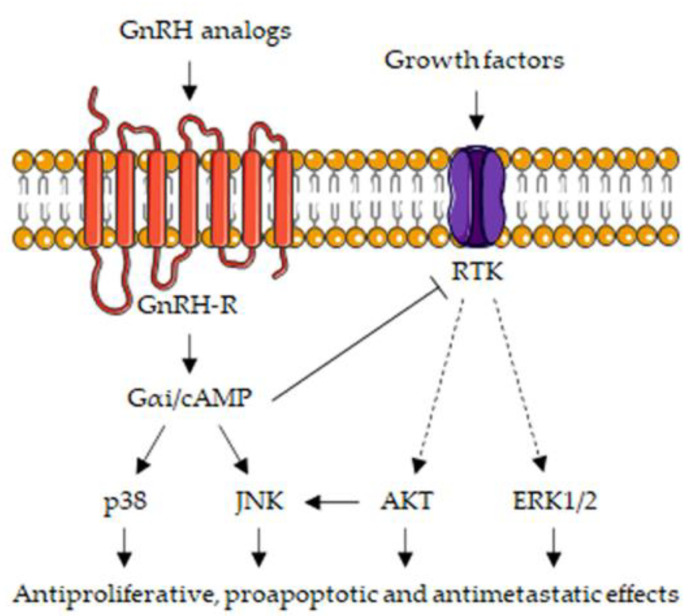
Intracellular signaling pathways involved in the antitumor activity of the GnRH-R in CRPC cells. The binding of GnRH analogs (both agonists and antagonists) to locally expressed GnRH-Rs triggers the activation of the Gαi/cAMP pathway. By decreasing cAMP levels, GnRH-R ligands activate the MAPK kinase cascades (i.e., p38MAPK and JNK), deeply involved in their anticancer effects. GnRH-R analogs also trigger the activation of a PTP, an enzyme responsible for the dephosphorylation (i.e., inactivation) of growth factor receptors (RTK), thus leading to the suppression of the expression/activity of their intracellular signaling mediators (i.e., ERK1/2 and AKT). In addition, AKT inhibition also leads to the stimulation of the JNK signaling pathway, further potentiating its antitumor activity. Abbreviations: cAMP, cyclic adenosine monophosphate; p38, p38 mitogen-activated protein kinase; JNK, c-Jun N-terminal kinase; RTK, receptor tyrosine kinase; AKT, protein kinase B; ERK1/2, extracellular signal-regulated kinase 1/2.

The main signaling pathways associated with the GnRH/GnRH-R axis in CRPC cells are summarized in Figure 3.

## 4. Androgen and Gonadotropin-Releasing Hormone Receptors: Molecular Targets for Therapeutic Strategies in CRPC

ADT, based on GnRH agonists or antagonists, still remains the mainstay treatment of hormone-dependent PCa. Results from different clinical trials also support the use of AR antagonists, such as enzalutamide and apalutamide, as well as of androgen synthesis inhibitors, such as abiraterone, for the treatment of hormone-sensitive PCa [4,244,245,246,247,248,249,250,251]. Unfortunately, in a few years, the majority of patients progress towards the CRPC stage, with tumor growing even in the presence of castration levels of circulating androgens. 

Taxane-based cytotoxic chemotherapy, i.e., docetaxel, is considered a therapy of choice for CRPC patients; cabazitaxel was also introduced in the clinical settings; however, its efficacy was found to be lower than that of docetaxel in a phase III clinical trial (FIRSTANA) [252]. Immunotherapy, such as sipuleucel-T or immune checkpoint inhibitors (nivolumab and pembrolizumab, binding and inactivating the T cell antigen PD1; durvalumab, targeting PD-L1), is another therapeutic strategy for CRPC patients. Unfortunately, serious side effects and a lower efficacy than expected are commonly associated with chemotherapy and immunotherapy, respectively [253,254,255,256,257]. 

Given the persisting role of the AR in the progression of PCa to the CRPC stage, inhibitors of the androgen pathways are commonly used for the treatment of CRPC patients [38,254,255,257,258]. Second-generation non-steroidal AR antagonists (enzalutamide, apalutamide, darolutamide) compete with androgens by binding to AR receptors and also inhibit AR translocation into the nucleus and its downstream binding to, and activation of, response elements in the promoter region of specific target genes. Enzalutamide was reported to significantly reduce PSA levels and the metastasis-free survival while increasing the progression-free survival (PFS) and the overall survival (OS) in non-metastatic and metastatic CRPC patients, either before or after chemotherapy [247,249,259,260,261,262]. Similar positive clinical outcomes were reported with the more recently developed, second-generation AR antagonists such as apalutamide and darolutamide [37,263,264,265]. Abiraterone, the inhibitor of CYP17A1 activity and, therefore, of androgen biosynthesis, demonstrated efficacy (reduction of risk of death, PFS and OS) and safety in metastatic CRPC patients, both in the pre- and post-chemotherapy settings [266,267,268]. Interestingly, it has been observed that, in a sequential treatment setting, starting therapy with abiraterone and subsequently switching to enzalutamide provides better results (in terms of PFS and PSA-PFS) than those obtained with the opposite sequence [39,255,257,269]. 

The presence of a GnRH/GnRH-R axis associated with antitumor activities in PCa and specifically in CRPC cells supports the notion that it might be considered an additional direct target of GnRH analog-based (agonists and antagonists) ADT. 

Gnanapragasam and coworkers reported that a high expression of GnRH-Rs correlates with a better clinical outcome in CRPC patients treated with GnRH agonists [174]. Moreover, it was shown that, in PCa patients, switching from a GnRH agonist (goserelin or leuprolide) to another agonist (leuprolide or goserelin), after disease progression, is associated with a reduction of PSA levels [270]. Co-treatment of chemotherapy-naïve CRPC patients with a GnRH agonist and docetaxel resulted in an improved PFS with respect to chemotherapy alone [271]. In line with these data, patients developing CRPC often continue on a GnRH agonist-based therapy when starting chemotherapy [272]. However, different results were reported by other clinical trials. In the ICELAND clinical trial, patients with advanced/relapsing PCa were treated with the GnRH agonist leuprorelin, either alone or in combination with bicalutamide. It was observed that continuous androgen deprivation did not improve PSA progression [273]. 

As discussed above, by binding to GnRH receptors expressed in PCa cells, as specifically in CRPC cells, GnRH antagonists behave as agonists triggering marked antitumor effects. To this purpose, it must be underlined that these compounds elicit a faster suppression of testosterone, as well as of FSH and PSA levels, in PCa patients [124,274]. Abufaraj and coworkers recently reported that, in patients with metastatic PCa, GnRH antagonists are associated with lower overall mortality rate (but without a significant difference in PSA progression) and cardiovascular events compared with GnRH agonists, while inducing higher injection site reactions [123]. Degarelix was shown to induce a more rapid decrease in testosterone levels and a better PSA control with respect to leuprolide in PCa patients [258,275,276,277]. Sugimura et al. showed that switching from a GnRH agonist to an antagonist (degarelix) was associated with a delay in tumor progression in a case of CRPC [278]; moreover, a recent systematic meta-analysis has pointed out that treatment with degarelix after failure of a GnRH agonist is associated with decreased or stable PSA levels in patients progressing to the CRPC phase [279]. However, in spite of these data, evidence to make an incontrovertible statement that GnRH antagonists have a greater efficacy than agonists in PCa treatment is considered still limited [123,280,281]. 

Recently, cytotoxic GnRH bioconjugates were developed as a new therapeutical approach for tumors expressing GnRH-Rs; these compounds consist of a GnRH-derivative covalently linked to a cytotoxic drug. It is expected that, by binding to its receptor, the GnRH derivative may specifically carry the cytotoxic drug to cancer cells without affecting normal cells. AEZS-108 (also known as AN-152), a bioconjugate consisting of a GnRH derivative covalently linked to doxorubicin via an ester bond, was developed, and its anticancer activity was investigated in different types of tumors [282,283,284]. This peptide cytotoxin was reported to exert a significant antiproliferative/proapoptotic activity in PCa, and specifically in CRPC cells, in vitro and in preclinical studies [285,286,287]. Results from phase I and II clinical trials demonstrated that, in chemotherapy naïve and taxane-resistant CRPC patients, AEZS-108 reduces PSA levels and is effective in increasing PFS [288,289]. Promising results were also obtained with different GnRH-based cytotoxic bioconjugates in CRPC cells, both in vitro and in vivo [283,290,291]. In our laboratory, we investigated the antitumor activity of bioconjugates consisting of daunorubicin linked to the GnRH-III isoform in CRPC cells. We found that these compounds are rapidly internalized and exert a significant antitumor activity in these cells [292]. Further studies are needed to confirm the efficacy and the lack of toxicity of GnRH-based cytotoxic bioconjugates in PCa. 

## 5. Conclusions and Future Perspectives

It is now well established that the progression of PCa towards its most aggressive CRPC phase is characterized by an active hormonal landscape.

Specifically, a persistent activity of the androgen/AR axis has been demonstrated to be present in CRPC cells. This is correlated to different molecular mechanisms, including: intratumoral synthesis of androgens; AR amplification; AR mutations; alternative splicing of the AR mRNA leading to the expression of AR splice variants (i.e., AR-V7 and Arv567es); increased expression/activity of transcription factors and coregulators of the AR. Based on these data, second-generation non-steroidal antiandrogens (enzalutamide, darolutamide, apalutamide) as well as inhibitors of enzymes involved in the androgen biosynthesis (abiraterone, the CYP17A1) are commonly used for the treatment of CRPC patients.

In addition, a GnRH/GnRH-R axis, widely reported to be associated with a substantial antiproliferative/proapoptotic, antimetastatic and antiangiogenic activity, is also expressed in CRPC cells. In line with these data, a high expression of GnRH-R in PCa tissues correlates with a better clinical outcome in CRPC patients. Interestingly, in cancer cells expressing the GnRH receptor, GnRH antagonists trigger the same antitumor activity of GnRH agonists, suggesting that differences in the molecular structure/mechanisms of these receptors might exist at the tumor vs. pituitary level. 

In accordance with these observations, ADT interventions, based on a gonadotropin-releasing hormone agonist, with or without an antiandrogen drug (AR antagonist or inhibitor of androgen synthesis), represent a mainstay treatment for PCa, even in the CRPC stage [255,257,258]. Recent clinical trials (PROSPER, SPARTAN, ARAMIS) reported a synergistic positive effect of AR antagonists (enzalutamide, apalutamide, darolutamide) and GnRH analog-based ADT on clinical outcomes (PSA levels and doubling time, median time to metastasis, PFS, OS, risk of death) in non-metastatic CRPC patients [262,293,294,295,296]. Results from these trials demonstrate that “initiating therapy at early stage of the disease is more effective than waiting until mCRPC development” [38]. The optimal sequencing and combination of these standard treatment strategies for CRPC patients is still a matter of debate [255,257].

In spite of these promising results, the incidence of PCa is still increasing. Underlying the molecular pathways of PCa development and progression towards the most aggressive, castration-resistant stage is mandatory for the identification of novel molecular markers/targets and therapeutic approaches for CRPC patients.

Assessing the AR status, and, specifically, the *AR* gene copy number, in plasma DNA is a minimally invasive tool for the identification of the development of resistance in PCa patients escaping enzalutamide- or abiraterone-based therapies. Recently, Beltran and coworkers reported that CRPC patients with high plasma *AR* copy number do not have a worse response to taxane-based chemotherapy compared with patients with normal plasma *AR*. These authors speculate that the analysis of plasma levels of *AR* copy number in the different stages of PCa might improve therapy selection for CRPC patients [297].

Several studies are presently ongoing with the aim to solve the issue of the role and subsequent potential targeting of AR mutations in PCa. As discussed in this review, specific AR mutations not only confer resistance to antiandrogens (such as enzalutamide) but can also convert these compounds in AR agonists, indicating a rapid development of drug resistance. In recent papers, by means of molecular docking and molecular dynamics simulations, novel AR antagonists were developed and found to exert a significant antitumor effect in CRPC cells as well as in PCa cells engineered to overexpressed the F876L mutant AR [298,299]. Moreover, a full characterization of AR mutations, achieved by genomic studies, will likely increase the treatment options for CRPC patients, in terms of personalized therapy. Recently, mutant *AR*s were reported to be easily detectable in cfDNA from CRPC patients [58,75,76,77]. It is now accepted that AR inhibition can be achieved also through its degradation. Selective AR degraders (SARDs) have been recently developed and demonstrated to exert significant antitumor activities in PCa [250,300]. However, further experimental studies as well as clinical trials are needed to confirm the utility of these novel approaches in the clinical setting.

AR-V7 is the most frequent AR splice variant detected in PCa, and, specifically, in CRPC tissues and its levels of expression, appears to be strictly correlated to resistance to enzalutamide and abiraterone. CTCs are now considered a clinically relevant biomarker of disease progression in different pathologies, including PCa. Recently, it has been reported that AR-V7 mRNA expression, assessed via the Adna test platform and the EPIC sciences CTC-based platform (an immunofluorescence-based detection assay that measures the AR variant protein localized in the nucleus), can be detected in the plasma from patients with mCRPC treated with enzalutamide. Compared with AR-V7 negative patients, AR-V7 positive patients treated with enzalutamide showed a shorter PFS and OS [301,302]. Thus, the assessment of the expression of different mutant/variant forms of the AR in CTCs is now considered an effective predictive biomarker of PCa progression and a useful tool for choosing the right therapeutic approach in terms of precision medicine.

Extracellular vesicles (EVs, previously called exosomes) carry different types of bioactive molecules to recipient cells in the tumor microenvironment. EVs mediate intercellular communications, including re-education of stromal cells, modulation of cancer metabolism, and also development drug resistance. The bioactive molecule profiles/signatures of tumor-derived Es change over time, reflecting the real-time status of cancer cells. Specifically, different bioactive molecules have been identified in PCa-derived EVs, such as AR and AR-V7, caveolin-1 (involved in cancer cell stemness), has-miRNA-940 (involved in the osteogenic differentiation of mesenchymal stem cells), integrin αvβ3 and αvβ6 (involved in PCa cell metastatic behavior), miR-409 (involved in prostate tumorigenesis), TGFβ (involved in the differentiation of mesenchymal stem cell into pro-invasive and pro-angiogenic myofibroblasts) and MDR-1 (multidrug resistance-1, involved in drug efflux) [95,303]. Based on these observations, PCa-derived EVs are now considered valuable diagnostic and prognostic biomarkers in progressing PCa, supporting their potential role in disease management.

As discussed in this review, GnRH receptors, associated with a significant antiproliferative activity, are expressed in PCa, and, specifically, in CRPC cells and tumors. Moreover, GnRH-R expression was also demonstrated in PCa stem cells isolated from explants of human prostate tumors. PCa patients expressing high levels of GnRH receptors, have a better clinical response to the GnRH-analog based therapy. GnRH analogs, either alone or together with AR inhibitors or taxane derivatives, still represent the mainstay therapy for CRPC patients.

Based on this observation, GnRH analog (GnRH, GnRH-II and GnRH-III)-based cytotoxic bioconjugates have been recently developed with the aim to specifically carry the cytotoxic drug (doxorubicin, daunorubicin) to cancer cells expressing the GnRH-R, while sparing normal cells. In particular, the bioconjugate AEZS-108 (AN-152) consists of a GnRH derivative linked to the cytotoxic drug doxorubicin. This compound was found to significantly inhibit the growth of CRPC cells, both in vitro and in vivo. Phase I and II clinical trials showed that AEZS-108 efficiently reduces PSA levels and increases PFS in CRPC patients. Further studies are needed to confirm these as promising. 

In conclusion, a deeper clarification of the expression and activities of both the androgen/AR and GnRH/GnRH-R axes in the CRPC stage will likely lead to the identification of novel predictive biomarkers as well as to the improvement of the therapeutical options for this almost untreatable disease, in terms of precision medicine.

## Data Availability

Not applicable.
